# The Prevalence of Hepatitis C Virus in Mashhad, Iran: A Population-Based Study

**DOI:** 10.5812/hepatmon.7723

**Published:** 2013-03-12

**Authors:** Mohammad Taghi Shakeri, Hosein Nomani, Majid Ghayour Mobarhan, Hamid Reza Sima, Sina Gerayli, Shirin Shahbazi, Sina Rostami, Zahra Meshkat

**Affiliations:** 1 Department of Biostatistics, Public Health School, Mashhad University of Medical Sciences, Mashhad, IR Iran; 2 Antimicrobial Resistance Research Center, Mashhad University of Medical Sciences, Mashhad, IR Iran; 3 Biochemistry of Nutritional Research Center, Faculty of Medicine, Mashhad University of Medical Sciences, Mashhad, IR Iran; 4 Department of Internal Medicine, Ghaem Hospital, Mashhad University of Medical Sciences, Mashhad, IR Iran; 5 Department of Biology, Faculty of Sciences, Ferdowsi University of Mashhad, Mashhad, IR Iran; 6 Women’s Health Research Center, Mashhad University of Medical Sciences, Mashhad, IR Iran

**Keywords:** Hepatitis C, Prevalence, Population Groups, Iran

## Abstract

**Background:**

Hepatitis C virus (HCV) infection is a significant health problem throughout the world. Chronic form of the disease is found in about 75% to 85% of the newly infected individuals. The chronic infection may lead to severe forms including chronic liver disease, cirrhosis and with a higher mortality rate, hepatocellular carcinoma. Since no vaccine has yet been developed against HCV, there is an increasing need to take measures to control the spread of the infection. Therefore, epidemiologic study of the virus is important to manage and monitor the spread of the virus in the community.

**Objectives:**

The aim of this study was to determine the prevalence of hepatitis C seropositivity in the general population of Mashhad, northeast of Iran.

**Patients and Methods:**

Three thousand, eight hundred and seventy (3870) individuals living in the city of Mashhad were recruited using cluster sampling method. HCV seropositivity was determined with HCV antibody detection ELISA kit and was confirmed by reverse transcriptase polymerase chain reaction (RT-PCR) method.

**Results:**

In this study the overall seroprevalence of hepatitis C was founded to be 0.2% by using ELISA method. However, the overall Hepatitis C virus infection prevalence was found to be 0.13% with RT-PCR method.

**Conclusions:**

Our study suggested that the prevalence rate of Hepatitis C virus is below 1% in the general population of Mashhad.

## 1. Background

Hepatitis C virus (HCV) is an enveloped, single-stranded RNA virus with positive polarity. This virus is one of the most important pathogens of the human and is able to cause mild to severe liver diseases. The virus is a member of the hepaciviruses genus in the Flaviviridae family. Hepatitis C virus is present in all regions of the world and about 170 million, which accounts for more than 3% of the world’s population, are infected. The virus can remain in the host for a long period. In 80% to 85% of the individuals infected with the chronic form of the disease the infection may lead to cirrhosis or hepatocellular carcinoma (1, 2). Because of the high prevalence of HCV in the world, it is critical to study the prevalence of the viral infection in each population to more effectively help health policy makers implementing programs to reduce the prevalence rate. The prevalence of hepatitis C varies throughout different regions of the world. A study conducted in Iran showed that the prevalence of hepatitis C virus among blood donors is very low (about 0.12% in 2002) (3). It seems that the prevalence of this virus in the general population of Iran is less than 1% (4), which is lower than the reports of the neighbor countries. It is 1.1% in Yemen, 0.9% among children and 1.8% among young generation in Saudi Arabia and 4% among blood donors in Pakistan (5). In a research conducted on blood donors in 1997 among 26 different provinces of Egypt, the prevalence was reported zero to 38% (6). In a study conducted in Japan from 2003 to 2007, the prevalence of Anti-HCV Ab was 0.15% among voluntary blood donors (7). In a study performed in Yemen in 2007, the prevalence of hepatitis C was found to be 0.8% among blood donors (8). Also, the prevalence of hepatitis C virus in particular patients with thalassemia and hemophilia, undergoing hemodialysis, and drug abusers has been determined. In a study in 2005, the prevalence of hepatitis C infection among patients with thalassemia of Amirkola area in Iran was reported to be 10.6%. This rate was much higher than voluntary blood donors in Iran (4). In 2007, the prevalence of hepatitis C infection in patients with thalassemia in Iran was reported 15.7% to 63.8% (9); while it was 32% among patients with thalassemia in Jordan in 2009 (10). Two studies were conducted on patients with hemophilic in Iran between 2007 and 2003. The prevalence rate of hepatitis C infection ranged from 15.6% (in Fars) to 76.7% (in western Azerbaijan) (11). According to the reports of Managing and Controlling Specific Patients Center, the prevalence of hepatitis C infection has decreased among patients undergoing hemodialysis from 14.4% in 1999 to 4.5% in 2005 in Iran (12, 13). In 2007, a systematic research was performed to show the prevalence of HCV among drug abusers and it was reported that in 49 of 57 countries, the rate of HCV positivity was about 50% among drug abusers (14).

## 2. Objectives

Since the prevalence of the virus infection had been studied only in certain groups in Mashhad, the authors decided to conduct this comprehensive study among the general population of Mashhad to evaluate its statistical prevalence.

## 3. Patients and Methods

In this study, 3870 individuals were employed from March 2010 to November 2011. Multistage Cluster Sampling method was used to ensure that the studied population represented the whole population of Mashhad. In the first stage, three classes were defined in Mashhad and in the next step; nine clusters within each center were defined by PPS2 method. Individuals were selected randomly according to the demographic information available from health centers of Mashhad. Samples were collected within blocks which were attributable to the standard statistical center of Iran. Following registration of 3870 in the study, informed consent was obtained and a questionnaire was filled for each participant. Ten milliliter of venous blood was taken as sample from the brachial vein and the sera were collected. The prevalence of anti-HCV Ab was determined by ELISA method using commercial kit (Delaware Biotech, USA). Positive samples were confirmed with reverse transcriptase polymerase chain reaction (RT-PCR). Briefly, the viral genome was extracted from samples using viral RNA extraction kit (Invisorb Spin Virus RNA mini kit, Germany) and HCV RT-PCR was performed using HCV specific commercial kit (STRP Hepatitis C Virus Detection Kit, CinnaGen Co., Iran). The PCR products were visualized on a 1.5% agarose gel by green viewer staining (Pars Tous, Iran) and UV photography. Data was analyzed with SPSS V.18 (SPSS, Chicago, IL) and Chi Square statistical test was used to evaluate the results.

## 4. Results

**Figure 1.**
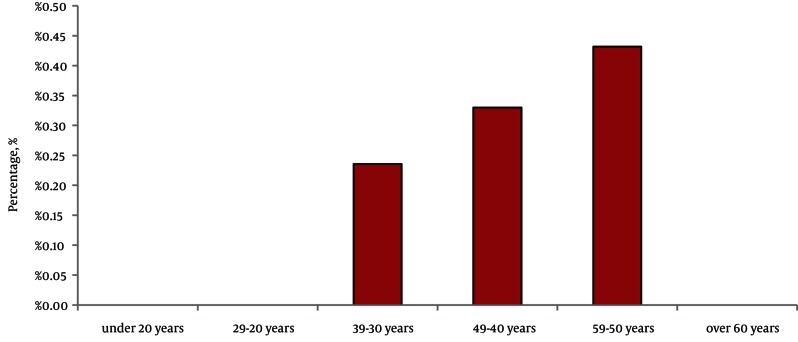
The Frequency of HCV Ab According to the Age Groups

**Figure 2.**
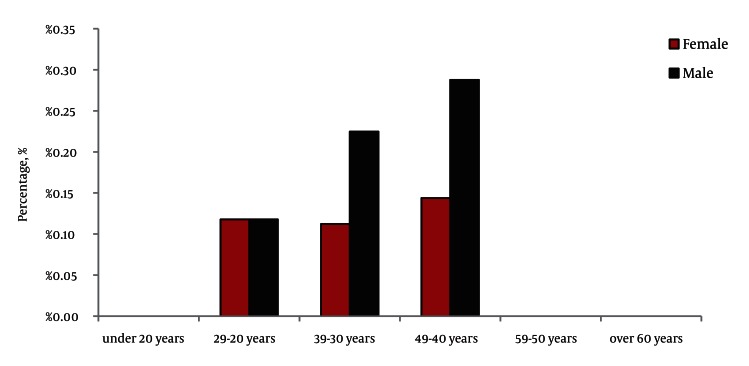
The Frequency of HCV Ab Among Male and Female According to the Age Groups

Our study was conducted on 3870 participants. It included 2589 (67%) female and 1281 (33%) male. The mean age was 39 years., The overall prevalence of positive hepatitis C antibody was 0.2% (8 of 3870 participants) using ELISA method, which included three female (0.11%) and five male (0.39%). The association between the HCV antibody positivity and gender was not statistically significant (*P* < 0.05). Overall, the prevalence of antibodies against HCV increased with older age and the highest prevalence rate was 0.43% in the age group of 50 to 59 years. In the age groups below 29 years and over 60 years, the rate of antibody positivity was zero (Figure 1). The highest prevalence of HCV antibody in women and men was 0.14% and 0.29%, respectively, in the age group of 50 to 59 years (Figure 2).

**Figure 3.**
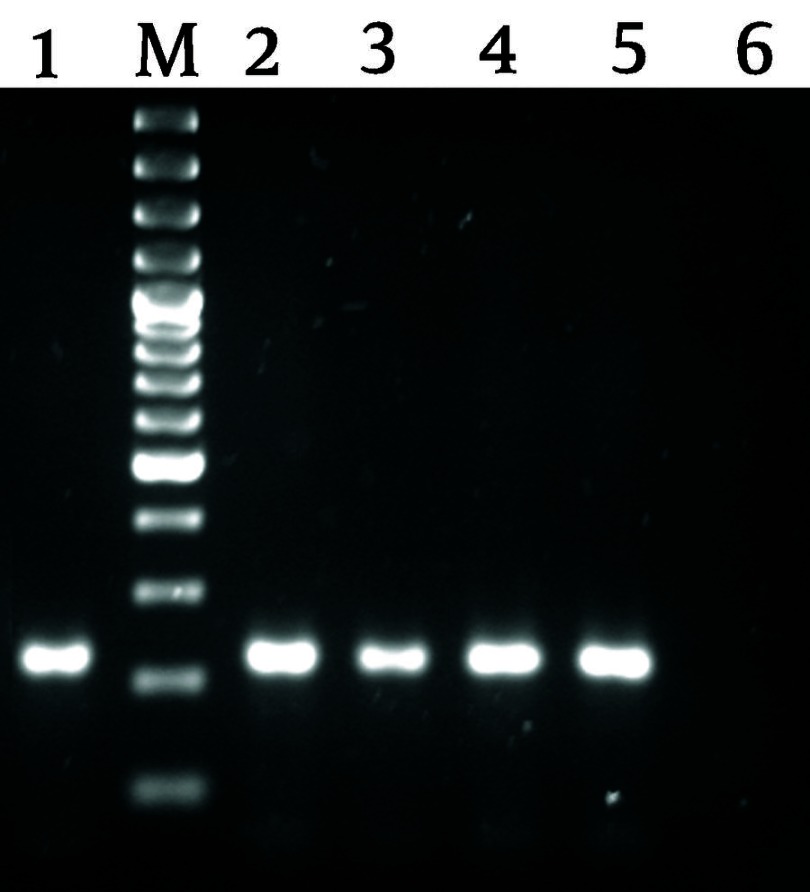
Gel Electrophoresis of RT-PCR Products RT-PCR was performed for amplifying a 216 bp fragment using HCV specific commercial kit. Lanes 1 and 6 are positive and negative controls of RT-PCR, respectively; lanes 2 to 5 correspond to a HCV positive RT-PCR for five positive sera; and M indicates a 100 bp DNA size marker.

Five of eight patients with positive result of the anti-HCV test had also positive results in RT-PCR method, in which a 216 bp band in agarose gel electrophoresis corresponding to the desired HCV specific band in our commercial kit was detected (Figure 3). The overall prevalence of hepatitis C infection which was confirmed by RT-PCR method was 0.13%. From five cases of hepatitis C infection confirmed by using RT-PCR method, 2 (0.07%) were female and three (0.23%) were male and the association was not statistically significant (*P* < 0.05).

## 5. Discussion

HCV prevalence studies in Iran are mostly focused on the high-risk groups such as injecting drug users, patients with hemophilia and thalassemia, and also patients undergoing hemodialysis. Since there is currently no effective vaccine to prevent hepatitis C infection, a large proportion of infected individuals are susceptible to develop cirrhosis and hepatocellular carcinoma (HCC). Consequently determining HCV prevalence is an important issue in the society. Numerous studies throughout the world have shown an increased incidence of HCV infection. Therefore, results of this study which concerns HCV prevalence in our city can be used for more effective decision making in the future. According to the Statistical Center of Public Health, Massachusetts (The United States) new reports of hepatitis C infection in the age group of 15 to 24 years indicated an increase from 65 to 113 in 100000 cases between 2002 and 2009. Drug injection is considered as the most significant risk factor. According to the Ministry of health in Iran, 1,200,000 people are infected with hepatitis B (HBV) and C viruses. However, according to unofficial reports, about two million are infected with HBV and between 200,000 to 300,000 are infected with HCV (15). In our study which was performed on 3870 participants who were living in Mashhad, HCV infection was detected with ELISA and was further confirmed by RT-PCR method. Figures indicated that the prevalence of antibody against Hepatitis C virus was 0.2% (eight positive cases) and the prevalence of confirmed hepatitis C with RT-PCR was 0.13% (5 of 8 cases). Prevalence of hepatitis C among blood donors in Iran in 2002, which was determined by ELISA method for anti HCV, was very low (about 0.12%) (3). the prevalence of hepatitis C antibody among blood donors of Yemen in 2002, which was determined by ELISA method, was reported 1.1% (16). However it reduced to 0.8% in 2007. In 1991, the prevalence of antibody against hepatitis C virus was reported to range between 0.9% among children to 1.8% among young generation of Saudi Arabia (17). Prevalence of hepatitis C, determined by ELISA method in sera of the blood donors in the north of Pakistan, was reported 0.4% in 2002 (5). Our study revealed that the prevalence of this viral infection is higher in our neighborhood countries regarding aforementioned investigations. Prevalence of antibody against hepatitis C virus, determined by ELISA method, was reported 0.15% among blood donors in Japan between 2003 and 2007 which was similar to our study (7). Although the reported prevalence rate is less than 1%, which is higher compared to our study (8). It seems that the prevalence of hepatitis C infection is less than 1% in Mashhad general population, which is lower than other countries. Throughout the world, injecting drug users account for about a half of all cases infected with hepatitis C. Injecting drug users are considered as potential reservoir of the virus in the society (9). The most common type of hepatitis is hepatitis C infection. Blood transfusion is a potential health threat in major thalassemia, hemophilia, and hemodialysis because they are dependent on the blood products during their lifetime. As a result, the expected prevalence of hepatitis C infection in these groups is higher than the normal population and blood donor volunteers. In 2007, the prevalence of hepatitis C infection in patients with thalassemia in Iran was between 63.8% and 15.7% (9). In a study performed on 122 patients in Jordan in 2009 found a prevalence rate of 32% among patients with beta thalassemia. As expected, the prevalence of hepatitis C infection was higher in studies which employed patients with thalassemia, unlike our study which was performed on the normal population. Patients undergoing hemodialysis are at an elevated risk compared to the general population for the prevalence and incidence of HCV infection. This, in turn, may affect the survival rate of the patients. According to the Managing and Controlling Specific Diseases Center, the prevalence of hepatitis C infection has been decreased among patients undergoing hemodialysis of the whole country from 14.4% in 1999 to 4.5% in 2005 (12, 13, 18). The prevalence of hepatitis C infection in patients with hemodialysis in the city of Sari in Iran was reported 18% in 2001 and 12% in 2006 (19). Prevalence of hepatitis C among patients undergoing hemodialysis was reported 5.4% in Tehran in 2005 (20). In Spain, in 2000, the prevalence of hepatitis C infection among patients with hemodialysis was reported 10.8% (21). However, the prevalence of the virus among patients with hemodialysis was 62.7% in Yemen in 2000 (8). As suggested by numerous studies in different parts of the world, the prevalence of hepatitis C infection in patients with thalassemia, hemophilia, and hemodialysis was higher than the normal population and also the similar one in our study. Regarding the report of Public Health Center of America (Massachusetts), hepatitis C infection rate has increased in the age group of 15 to 24 between 2002 and 2009. In this age group, similar to our study, sexual distribution of the infection was the same in both genders. However, in our study, the frequency of hepatitis C was zero among patients under 29 years old and the highest prevalence was in the age group of 50 to 59. A better understanding of the pattern of hepatitis C prevalence would be helpful to minimize its national and global prevalence. Several measures have been suggested to reduce its spread which includes but is not limited to distribution of informative pamphlets about risk factors and necessity of safe blood transfusion among the general public; encouraging the educated groups for more blood donations, which may contribute to reduction of HCV, HBV and HIV prevalence in the donated blood, because this group of people have a better perception of informing materials and they may refuse to donate blood if they are at risk of infection; increasing the voluntary counseling and guiding activities, which may lead to fewer donors who need to be examined and finally improvement of general health programs, focusing on counseling and screening of the subjects participated in high risk activities. The availability of cheap but sensitive and specific tests for determining the infection of HCV would be of great importance in the reduction of its transmission. Moreover, common needles injections among patients with addiction must be prevented and the pattern of addiction should be modified to a less harmful one.
